# Agl, The Multitasking Motor Protein

**DOI:** 10.1371/journal.pbio.1001729

**Published:** 2013-12-10

**Authors:** Mary Hoff

**Affiliations:** Freelance Science Writer, Stillwater, Minnesota, United States of America

[Fig pbio-1001729-g001]It slices, it dices, it cleans the kitchen sink. We've all heard of—and hailed the ingenious efficiency of—devices that perform more than one function. But humans don't have the corner on that market. A team led by Tâm Mignot and Morgane Wartel recently discovered that when the going gets rough, a common soil-inhabiting bacterium, *Myxococcus xanthus*, repurposes a molecule that's integral to the mechanism it uses to move around to build a fortress-type coat that protects it from its newly adverse environment.

**Figure pbio-1001729-g001:**
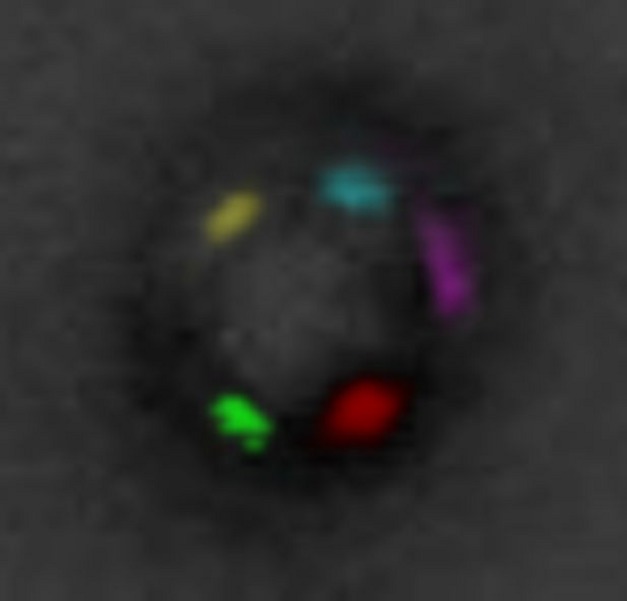
The Agl motor caught in action: positions of Nfs clusters around a spore at different times are shown in distinct colors.

The story behind the discovery of this remarkable example of making the most of what you have began several years ago, when the team uncovered the mechanism *M. xanthus* uses to move across a solid surface. They found that a molecular motor, Agl, combines with a second molecular complex, Glt, forming an assembly that the bacterium uses to propel itself with the help of a sugar polymer they call slime. Further analyzing this system, the researchers realized that the genes used as a blueprint to make Glt are similar to—and likely derived from a common ancestor gene with—those used to make another protein complex, Nfs, which must be present in order for the bacterium to use another sugar polymer, Exo, to form a hard, protective coat around itself when it encounters adverse environmental conditions. Considering the similarities between Glt and Nfs and the fact that they both used complex sugars to do their job, the researchers wondered: Might Agl be a multipurpose molecular device, deployed in the Nfs system in a similar manner to the way in which it interacts with Glt, when the cell's task turns from moving around to taking cover within its self-contained defense system?

To answer that question, the researchers took a closer look at how Nfs does its job. By testing the capabilities of mutants lacking the ability to make various proteins, they were able to assess whether genes that code for a different Agl-like molecule are required for spore formation (they aren't) and whether Agl is required for spore coat assembly (it is). Knowing that the Agl complex connects with Glt to create the motility function through the association of a helper molecule, GltG, the researchers then looked for and found a similar helping molecule that is required for proper Nfs system functioning. On the basis of this evidence, they concluded that the same Agl complex involved in motility does indeed associate with Nfs to provide the spore-forming function.

How exactly does the Agl-Nfs complex create a shell strong enough to protect *M. xanthus* when times get tough? To answer that question, the researchers used special molecular tools and analysis of the capabilities of various *M. xanthus* mutants to observe, and in some cases deduce, the movement and activities of Agl, Nfs, and Exo as they work together to form a spore coat. The various analytical approaches indicated that the process likely begins when, in the face of adversity, the genes used to make the “gliding” molecular complex Glt turn off and those for the “necessary for sporulation” Nfs molecular complex turn on. As Nfs is formed in the wake of activation of its corresponding gene, it spreads across the outside of the bacterial cell, transported by Agl and most likely guided by Exo, which is exported from within the cell at a few select locations on the cell surface. As it spreads, the Nfs-Agl complex assembles the protective spore coat by creating a meshwork of Exo strands anchored to the surface of the cell.

The researchers concluded that Agl does indeed play a role in two distinct but remarkably parallel functions within *M. xanthus*, with the distinction between them dependent on the nature of the accompanying molecular complex, which in turn depends on whether the cell is in move-about or duck-and-cover mode. Reflecting their common genetic heritage, Agl-Glt and Agl-Nfs perform broadly similar functions in transporting materials across the surface of the cell—in the former case, motility-empowering slime, and the latter, the threads that together weave a tight, protective shell. When *M. xanthus* goes into spore-forming mode, Agl breaks off from Glt and latches onto Nfs, where it serves as a transporter, distributing Nfs around the cell surface in a way that allows it to engage Exo to form the shell.

The authors note in closing that complexes similar to Agl exist in other kinds of bacteria, inviting exciting future studies of potential transport functions in other systems. They also observe that the fact this one system, with slight modification, performs two quite different functions, opens the door to a new view of how minor changes at the genetic level can usher in dramatic new capabilities for living systems.

**Wartel M, Ducret A, Thutupalli S, Czerwinski F, Le Gall A-V, et al. (2013) A versatile class of cell surface directional motors gives rise to gliding motility and sporulation in** ***Myxococcus xanthus.*** doi:10.1371/journal.pbio.1001728

